# Supplementary material for: Evaluating the oestrogenic activities of aqueous root extract of Asparagus africanus Lam in female Sprague-Dawley rats and its phytochemical screening using Gas Chromatography-Mass Spectrometry (GC/MS)
Source: PeerJ. 2019 Jul 19;7:e7254. doi: 10.7717/peerj.7254 (PMC6644626; doi:10.7717/peerj.7254)
Supplement: Supplemental Information 2 [file peerj-07-7254-s002.pdf]

The mass spectrum of all 9 compounds are presented as supplementary materials and labelled from figure S1 to figure S9.

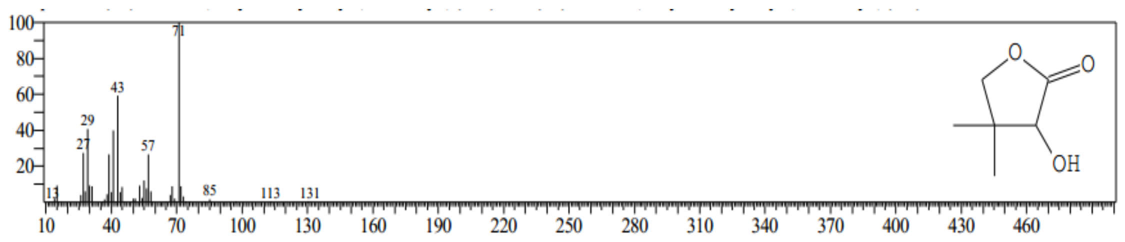

Figure S1: Mass spectrum and chemical structure of (3H)-Furanose, dihydro-3-hydroxy-4,4-dimethyl

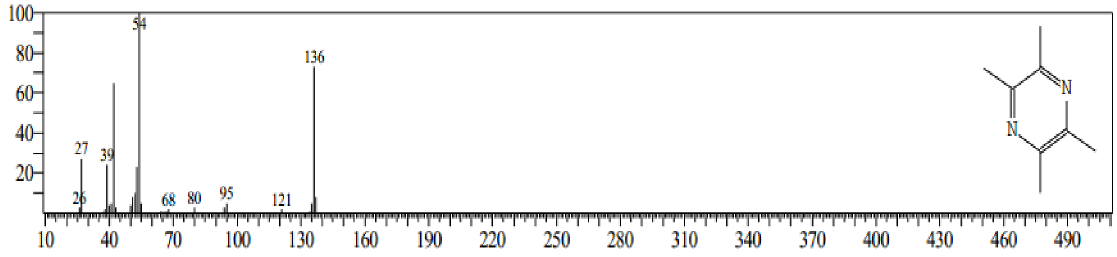

Figure S2: Mass spectrum and chemical structure of Pyrazinetetramethyl

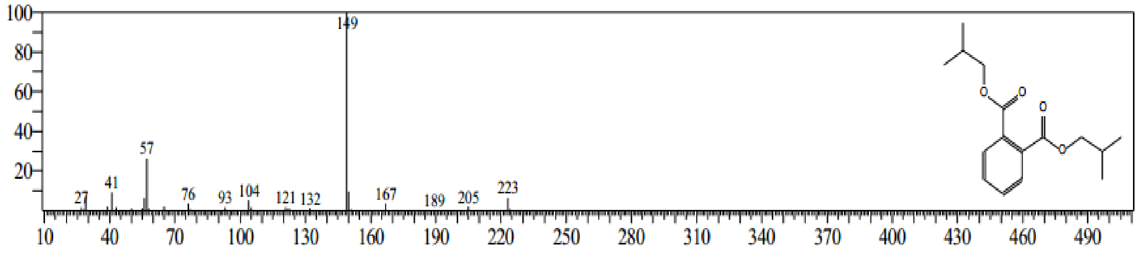

Figure S3: Mass spectrum and chemical structure of 1,2-Benzenedicarboxylic acid

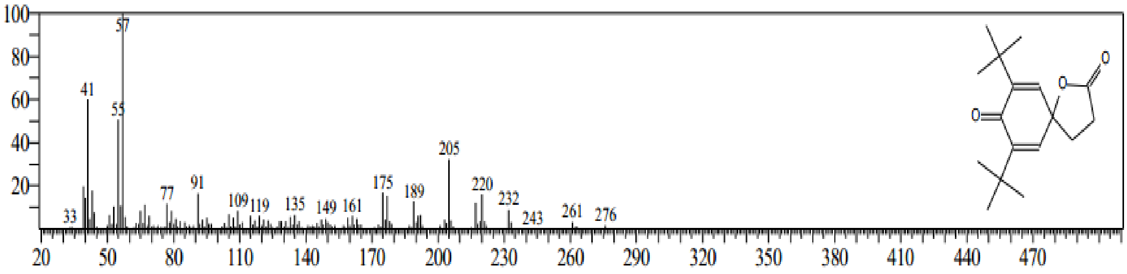

Figure S4: Mass spectrum and chemical structure of 7,9-Die-tert-butyl-1-oxospiro (4,5) deca-6,9-diene-2,8-dione

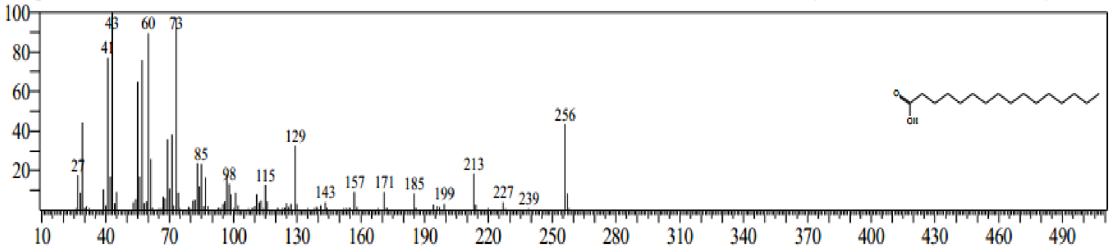

Figure S5: Mass spectrum and chemical structure of n-Hexadecanoic acid

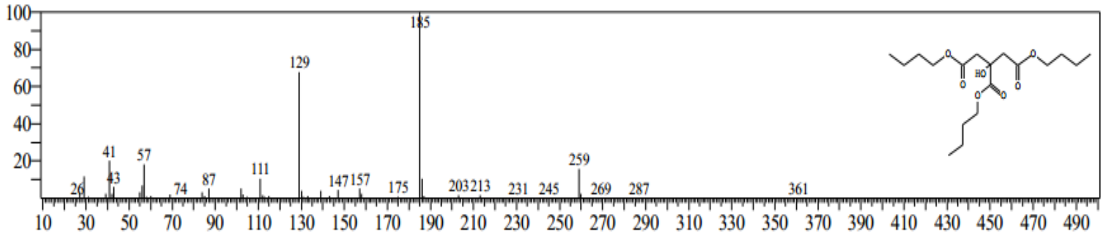

Figure S6: Mass spectrum and chemical structure of 1.Butyl citrate

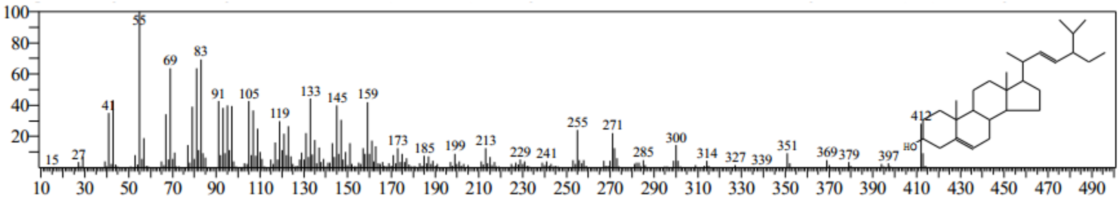

Figure S7: Mass spectrum and chemical structure of Stigmasterol

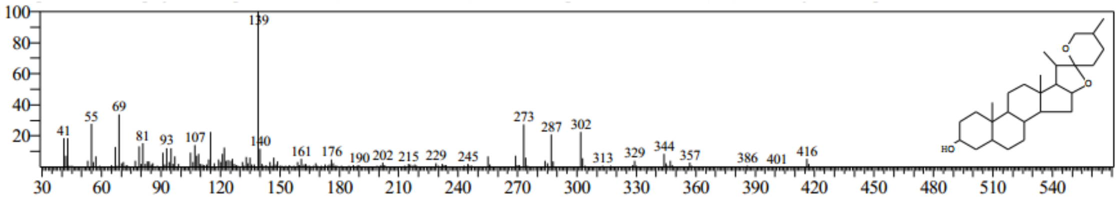

Figure S8: Mass spectrum and chemical structure of Sarsasapogenin

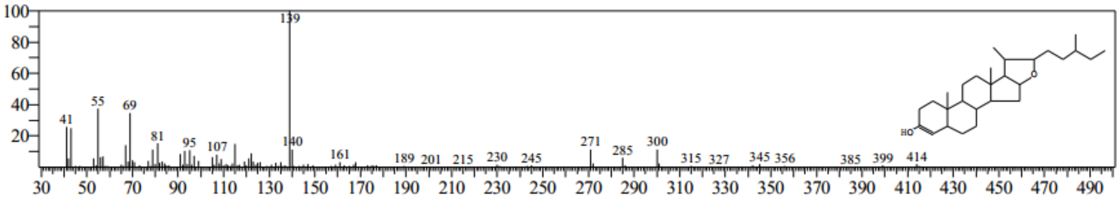

Figure S9: Mass spectrum and chemical structure of 3-Dehydro-des-N-26-methyl-dihydro-pseudotomatidine
